# Lymphomatoid granulomatosis involving the central nervous system: A case report and review of the literature

**DOI:** 10.3892/ol.2014.2002

**Published:** 2014-03-28

**Authors:** HONGLI LIU, JING CHEN, DANDAN YU, JIANLI HU

**Affiliations:** Cancer Center, Union Hospital, Tongji Medical College, Huazhong University of Science and Technology, Wuhan 430022, P.R. China

**Keywords:** lymphomatoid granulomatosis, lymphoma, chemotherapy

## Abstract

Lymphomatoid granulomatosis (LYG) is a rare tumor with unknown etiology. Specific etiological factors for LYG are also unknown, although previous data indicates that LYG is an Epstein-Barr virus-associated B-cell proliferation associated with an exuberant T-cell reaction. According to the 2008 WHO classification, LYG is characterized by B-cell proliferation of B-lymphoma cells. Generally, treatment options for LYG are similar to those for diffuse large B-cell lymphoma. Unfortunately, LYG is a chemotherapy-resistant disease in certain patients and has a poor prognosis. The current study presents the case of a 19-year-old male patient with pulmonary LYG. The patient exhibited progressive disease following one cycle of chemotherapy with cyclophosphamide, adriamycin, vincristine and prednisone, and nodular lesions in the brain were diagnosed. Radiotherapy was delivered to the whole brain, however, this treatment did not prevent progression of the disease and the patient succumbed three months after initial presentation. An overview of the literature with regard to the etiology, clinical features, diagnosis and treatment options for LYG is also presented in the current case study.

## Introduction

Lymphomatoid granulomatosis (LYG) is a rare Epstein-Barr virus (EBV)-associated disorder and is considered part of the spectrum of lymphoproliferative disorders. While commonly presenting with multiple lung nodules, in occasional cases, atypical patterns can be observed radiographically. Specific systemic manifestations can occur, including fever, weight loss, skin lesions and the involvement of other organs (1). The clinical presentation of LYG can mimic infectious diseases (including tuberculosis), vasculitis or metastatic malignancies. According to the 2008 WHO classification, LYG is characterized by the proliferation of B-lymphoma cells (2). Patients may present with non-specific neurological symptoms, including seizures and incontinence (3). In order to make a definitive diagnosis, biopsies, pathological examinations and immunohistochemical analyses must be performed. In the current study, an overview of the literature in reference to the etiology, clinical features, diagnosis and treatment options for LYG is presented. Patient provided written informed consent.

## Case report

A 19-year-old male was presented to the pulmonary clinic of the People’s hospital of Shenzhen University (Shenzhen, China) with chills, a fever, mild intermittent dry coughing, night sweating and fatigue that had lasted 7 days. One month prior to this presentation, the illness began with a low fever, mild coughing and fatigue. The patient was treated symptomatically for a presumed upper-respiratory viral infection. The patient had fevers as high as 40.2°C and had no significant exposure history. The patient had no risk factors for human immunodeficiency virus (HIV) infection, and was an ex-smoker.

Upon presentation, the patient did not experience respiratory distress and the vital signs were as follows: Temperature, 40.2°C; heart rate, 110 beats/min; respiratory rate, 24 breaths/min; and blood pressure, 120/70 mmHg. Oxygen saturation (measured via digital pulse oximetry) was 95% while breathing ambient air. The skin was diaphoretic and there were no palpable lymph nodes in all areas. Chest, cardiac, abdominal and skin examinations did not reveal abnormalities. On lung auscultation, breath sounds were diminished over the lower left lung, and there were no crackles or rales on either side. A neurological examination revealed no focal defect.

**Figure 1:**
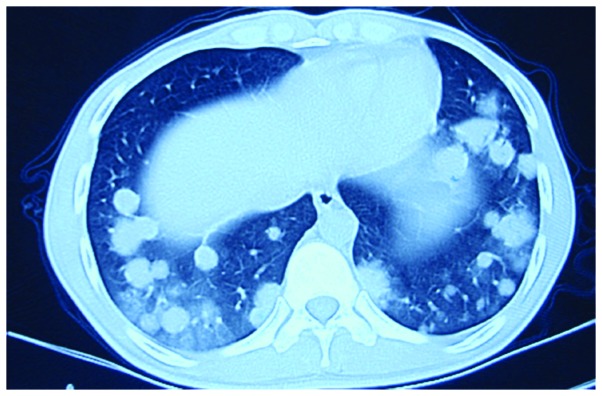
Computed tomography (CT) image demonstrating multiple pulmonary nodules.

**Figure 2:**
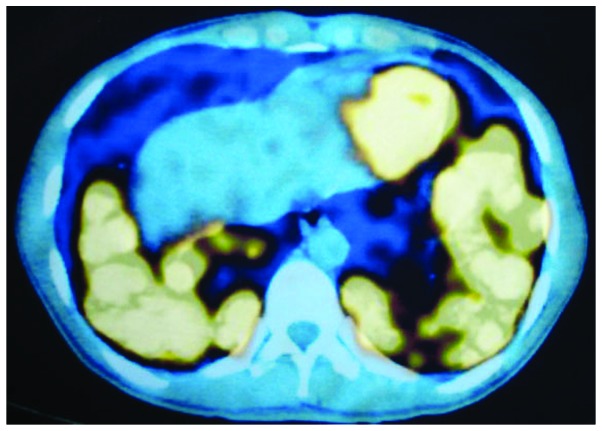
Computed tomography-positron emission tomography (CT-PET) image demonstrating multiple hypermetabolic pulmonary nodules.

**Figure 3:**
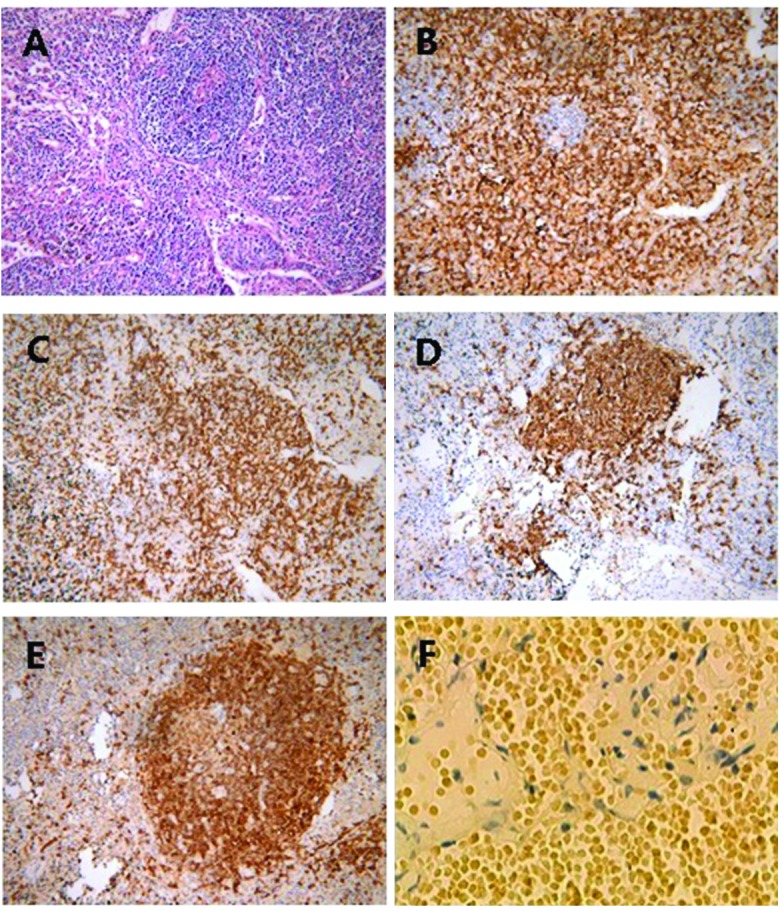
Histological features of a pulmonary nodule biopsy. (A) HE staining demonstrating an angiocentric and angiodestructive infiltrate of lymphoid cells in the vascular wall, with surrounding infarct-like tissue necrosis (x200). Immunohistochemically, the infiltrates were positive for (B) CD45RO, (C) CD3, (D) CD20 (medium and large leukocytes) and (E) CD79α (x200). (F) Epstein-Barr virus (EBV) expression was strongly positive in the lymphocytes, as observed by *in situ* hybridization analysis (x200).

The white blood cell count of the patient was 4.7×10^3^/dl and the hemoglobin level was 13.0 g/dl. Serum electrolyte levels and liver and renal function were normal. Spirometry values and the erythrocyte sedimentation rate (ESR; 7 mm/h) were normal. Routine blood and sputum cultures for detection of acid-fast bacteria were negative. Serological tests revealed that there was no infection with hepatitis B virus, hepatitis C virus, HIV and tubercle bacillus. Computed tomography (CT) imaging demonstrated bilateral pulmonary multiple round nodules, predominantly in the lower lung fields, along the bronchoalveolar structures (Fig. 1). There was no mediastinal or hilar lymph node enlargement. F-18 fluorodeoxyglucose (FDG) positron emission tomography (PET) revealed multiple hypermetabolic pulmonary nodules in the lungs, predominantly in the lower lobes [maximum standard uptake value (SUVmax), 3.3–7.8; Fig. 2). Splenomegaly was observed and the SUVmax was 12.4 in the PET/CT image. The CT-guided transthoracic biopsy revealed inflammatory cells and large coagulated necrotic tissue. The patient consented to the removal of a thoracoscopic biopsy, which revealed an angiocentric and angiodestructive infiltrate of lymphoid cells in the vascular wall, with surrounding infarct-like tissue necrosis (Fig. 3). Immunohistochemical staining revealed that the lymphoid infiltrate consisted of a mixture of cells expressing cluster of differentiation (CD)45RO, CD3, CD20 and CD79α. Furthermore, *in situ* hybridization for the detection of EBV was strongly positive (Fig. 3). A diagnosis of LYG was finally established.

**Figure 4:**
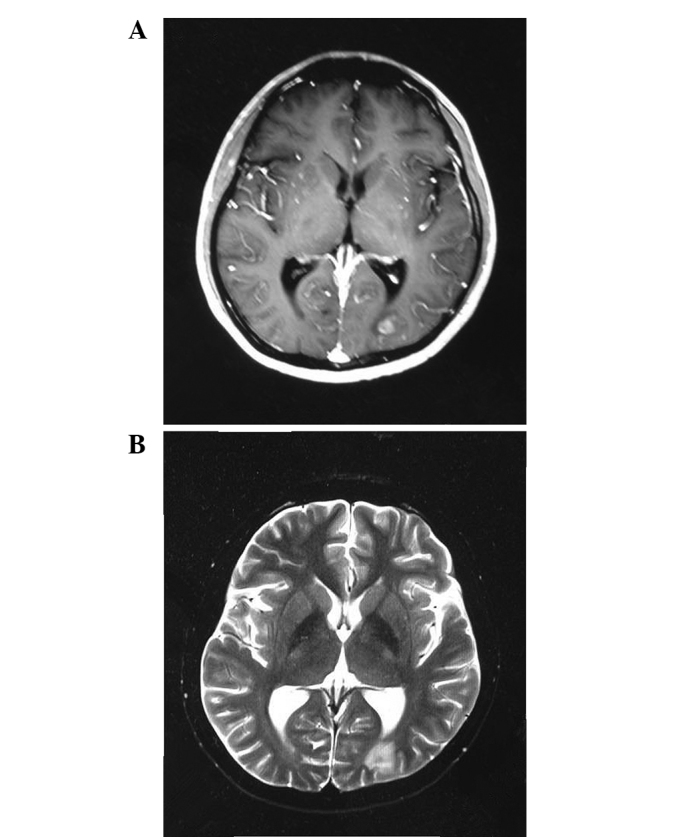
(A) T1- and (B) T2-weighted brain magnetic resonance (MR) images demonstrating multiple irregular and nodular lesions with peripheral edema in the bilateral parietal lobe and the left cornu occipitale of the substantia alba.

The patient received chemotherapy with cyclophosphamide, adriamycin, vincristine and prednisone (CHOP). The body temperature was normal during the chemotherapy. However, only two days subsequent to the end of chemotherapy, the patient developed a generalized tonic-clonic seizure and fever. T1- and T2-weighted magnetic resonance (MR) images revealed multiple irregular and nodular lesions, with peripheral edema in the bilateral parietal lobe and the left cornu occipitale of the substantia alba (Fig. 4). Central nervous system (CNS) involvement was considered. Therefore, the patient received whole brain irradiation with a total dose of 30 Gy in 10 fractions. This treatment did not halt the generalized tonic-clonic seizure, while the coughing, shortness of breath and hyperpyrexia continued. A chest-CT revealed a left-sided pleural effusion, lobus inferior pulmonis atelectasis and small amounts of pericardial effusion. The patient received 2 l/min oxygen by nasal canula. Subsequently, the patient developed rapid pulmonary failure, and succumbed ~3 months after presentation due to progressive pulmonary and CNS disease.

## Discussion

LYG is a rare lymphoproliferative disease. Liebow *et al* designated the term ‘lymphomatoid granulomatosis’ almost 40 years ago, as an angiocentric and angiodestructive lymphoreticular proliferative and granulomatous disease with EBV infection, lung lesions and a poor outcome (4). The first description of the disease was followed by several reports about the nature, histopathology and treatment of LYG. The following is an overview of this literature.

LYG has an increased male predilection, with male-female ratios ranging between 2:1 and 3:1. Generally, patients are between 40 and 60 years old (5). LYG frequently presents as a systemic disease, usually with prominent pulmonary sites. Other sites can also be involved, including the skin, CNS, kidney, liver, upper respiratory tract and gastrointestinal tract. Lymph node and spleen involvement are less common (7–8%). Constitutional symptoms, including weight loss, fatigue, sweating and chills, are typical (6). Local symptoms include coughing, shortness of breath and chest pain, while almost one-third of patients develop neurological symptoms, including confusion, ataxia, hemiparesis or seizures, mainly due to lesions in the CNS. Cranial nerve palsies and peripheral polyneuropathy have also been described in 7% of cases (7–10).

Chest X-rays reveal bilateral lesions in 71–90% of patients. The most frequent observations are multiple nodules or masses with poorly defined margins, whereas reticular or nodular infiltrates are less often described. Cavitations or solitary masses are rarely presented. Pleural effusions (25%) are usually small and present as costophrenic angle blunting. Mediastinal lymphadenopathy is visible on CT in 60% of patients, and nodules are characteristically observed alongside bronchovascular structures and interlobular septa (11,12). The application of FDG-PET for tumor imaging has proved to be highly useful for the diagnosis of primary lung tumors. If LYG is radiologically proven to involve the CNS, this is shown as multiple punctate and linear enhancement on CT and MR images, representing lesions in perivascular tissue and the walls of small blood vessels (13). Patsalides *et al* (14) conducted a comprehensive retrospective analysis of the MRI results of 25 patients with LYG and found a wide spectrum of CNS lesions in 52% (13/25) of the patients. In the most frequent cases, the patients presented with multifocal intraparenchymal brain lesions, which exhibited punctate or linear enhancement (n=7), followed by leptomeningeal and cranial nerve involvement (n=6).

LYG is similar to Wegener’s granulomatosis in clinical features and imaging, including pulmonary manifestations and the systemic nature. The differential diagnosis includes tuberculosis, granulomatosis, lung cancer and inflammatory pseudotumors. The prognosis of LYG is extremely variable, ranging from extremely good in the cases of spontaneous resolution, to rapidly fatal. The median survival time ranges between 14 months and 4 years, and the 5-year mortality rate is 60–90%. In total, 94% of mortalities occur in the first 36 months following diagnosis (15–17). Certain studies indicate that leucopenia, fever, anergy in reaction to common skin test antigens, a young age and localization in the CNS are poor prognostic signs (7,8,18). A biopsy must be taken to make a definitive diagnosis. Transbronchial biopsy is not recommended, as it is diagnostic in only 27% of cases, while open lung biopsy specimens are uniformly positive (19).

As LYG is a rare disease, a standard treatment paradigm has not yet been established, and consequently, the treatment of LYG remains a challenge. Certain patients have self-limiting disease, a number are treated with corticosteroids, either as single agent or combined with cyclophosphamide, and certain patients are treated with another chemotherapy, including CHOP or COP regimens, depending on severity at presentation. LYG responds poorly to conventional chemotherapy, and no significant differences have been identified between the regimens, while radiotherapy has also been used for CNS and orbital localizations (7,20,21). Rituximab-CHOP, or other similar regimens, are usually recommended for treatment of grade 3 LYG due to the high rate of rapid progression into EBV-positive large B-cell lymphoma (22,23). Several case studies have been published in which treatments with interferon α-2b, with or without autologous stem cell transplantation (24–26), and prednisone, methotrexate, doxorubicin, cyclophosphamide, etoposide, mechlorethamine, vincristine, and procarbazine or cyclosporin-A were presented (27). For the patient in the present case study, CHOP chemotherapy and radiotherapy failed, and rapid progressive disease was observed in the CNS. The present case demonstrated that LYG is a chemotherapy-resistant disease in certain patients. LYG remains rarely recognized as an entity, and proper diagnosis and treatment remain a challenge.
